# Community health workers lensed through a South African backdrop of two peri-urban communities in KwaZulu-Natal

**DOI:** 10.4102/ajod.v6i0.294

**Published:** 2017-08-29

**Authors:** Meghan S. White, Pragashnie Govender, Helga E. Lister

**Affiliations:** 1 Department of Health, University of KwaZulu-Natal, South Africa; 2 School of Health Sciences, University of KwaZulu-Natal, South Africa

## Abstract

**Background:**

As the South African government re-engineers primary healthcare (PHC), the need for additional information on stakeholders involved in the process has emerged. Of these are community health workers (CHWs), who have been identified as central to PHC success.

**Objectives:**

To profile the current CHWs within KwaDabeka and Clermont in KwaZulu-Natal, to describe their roles and to explore the barriers and enablers influencing their service delivery.

**Method:**

A convergent mixed methods design was employed with a sample of CHWs with the use of a survey (*n* = 53) and two focus groups (*n* = 10) and semi-structured interviews with four ward councillors (*n* = 4). Data were analysed statistically and thematically.

**Results:**

The profile of CHWs reflected only women with a mixed age range and a majority of 59% who had not completed formal schooling. General work experience as a CHW varied. There were diverse opinions expressed towards the CHW role which related to their job title and identity, supervision, remuneration, growth pathways and psychological and emotional issues. Whilst the National Community Health Worker Profile Framework was established for the CHW programme, there are several factors lacking in the current CHW programme such as a formal growth pathway or formal training to align the CHWs with the National Qualifications Framework.

**Conclusion:**

The study findings are essential for the monitoring and evaluation as well as development and refinement of policies that will assist in ensuring adequate rollout of PHC with CHWs.

## Introduction

The World Health Organization (WHO) has identified a chronic global shortage of well-trained health workers, creating an essential need for sustainable development strategies for health systems (WHO 2006).

Primary healthcare (PHC) is part of South Africa’s sustainable development plan to establish equitable health and healthcare services. The principles of PHC are evident in policy development in South Africa from as early as the 1900s, including the National Health Services Commission (NHSC or Gluckman Report) (Republic of South Africa 1945) and the African National Congress’s (ANC) health planning prior to 1994 (ANC 1994). The ANC provided the groundwork for the post-1994 health sector transformation, and recommendations developed at the 1978 Alma Ata conference on PHC were included in policy, thus began a country-wide PHC service rollout.

Currently, an estimated 5482 PHC outreach teams service the uninsured^1^ population of South Africa and these teams each need to reach 84% of the total population of South Africa, who are based in rural areas, informal urban settlements and townships (Department of Health [DOH] 2011b).

### Community health workers in South Africa

Key members of the PHC outreach teams are community health workers (CHWs), defined as ‘people chosen within a community to perform functions related to healthcare delivery, who have no formal professional training or degree’ (Van Ginneken, Lewin & Berridge 2010:1110). CHWs screen, map, educate, link and extend PHC in the communities for which they are responsible (Networking HIV 2013). CHWs provide services to communities, families and individuals at community-based institutions and also at a household level in each municipal ward (Ngcwabe & Govender 2013).

Considering the importance that CHWs play in the rollout of PHC in South Africa, an investigation into the current CHWs in preparation for their participation in the rollout is necessitated. In most South African districts, there is a lack of knowledge of the demographic profile of CHWs as well as documentation around the efficacy of service delivery. This is re-iterated by Ngcwabe and Govender (2013) who argue that CHWs cannot fulfil their role as members of the Ward-Based PHC Outreach Team (WBPHCOT)^2^ if they have not received appropriate education and training, and if they are not able to function at the required level of competence. Taking the lead from international policy, South Africa now strives to roll out a comprehensive PHC programme, and thus direct knowledge of current CHWs and their experiences will be essential to guide the process of change in South Africa. More importantly, it will also play a role in assisting the various stakeholders involved in healthcare policy guideline development to design policies that may be clearer to implement and thus more effective.

### Pertinent policies, frameworks and acts pertaining to the community health worker programme

Several policies have been developed in light of the CHW programme; however, the National Qualifications Framework^3^ (NQF), Expanded Public Works Programme (EPWP) (Department of Public Works 2017), the National Community Health Worker Policy Framework (NCHWPF) and *Basic Conditions of Employment Amendment Act*, No. 11 of 2002 (South Africa 2002) are some of the most pertinent to CHWs. These policies govern the CHW programme; however, contradictions and policy gaps were noted. Table 1 depicts the frameworks and how these govern important aspects of the CHW programme specifically.

**TABLE 1:** Pertinent frameworks in relation to the community health worker programme.

Policy area	NQF	EPWP	NCHWPF	BCEA
**Date of inception or implementation**	2008	2004	2004	2002
**Stipend**	Not applicable	CHWs are paid an allowance. R500 for NQF Levels 1, 2, 3 R1000 for NQF Level 4 qualification	CHWs to receive a stipend. Amount and pay point differs from province to province.	Reported hours that CHWs work indicate that they should be salaried. The amount should be aligned to a national standard.
**Formal growth pathway**	Growth pathway is achieved by completing NQF levels.	The EPWP will up-skill volunteers without Matric to NQF Level 3 and those with Matric up to NQF Level 4.	Volunteers were absorbed into the programme as Generalist CHWs. There is no current growth pathway for CHWs to qualify with a higher rank.	Not applicable.
**Conditions of employment**	Not applicable	Conditions stipulated in job offer and work contract. Work 40 h in a 5 day week, but only earn a stipend that is dependent on the province they work in and not a national standard.	Conditions stipulated in job offer and work contract. Work 40 h in a 5 day week, but only earn a stipend that is dependent on the province they work in and not on a national standard.	National standard is established, to which all employers must adhere to.
**Formal job title and status**	NQF L1: Ancillary Health Worker NQF L2&L3: Community Care Worker NQF L4: Community Health Worker	Community Health Worker is used as formal title, and individuals are considered as employees. Individuals can volunteer and are unpaid for the programme.	Community Health Worker is the formal title, but there are still other titles such as ‘Community Care Giver’, ‘Home-Based Carer’ and ‘uNompilo’ being used. Individuals can volunteer and can be unpaid for the programme. CHWs are considered employees.	Not applicable.
**Supervision**	Not applicable	CHW supervisors are allocated a team of CHWs.	An ‘Outreach Nurse’ fulfils both the role of a nurse and CHW supervisor at the CHC. A ward councillor also supervises CHWs.	Not applicable.

NQF, National Qualifications Framework; EPWP, Expanded Public Works Programme; NCHWPF, National Community Health Worker Policy Framework; BCEA, *Basic Conditions of Employment Amendment Act.* No. 11 of 2002; CHW, Community Health Worker; CHC, Community Health Centre.

In this article, the authors thus seek to provide a snapshot of the CHW programme in two communities of KwaZulu-Natal (KZN) that was part of a pilot study into CHWs. It provides an opportunity to examine the out-workings of the implemented policies governing the CHW programme by providing information around potential factors that impact service delivery.

## Research method and design

### Study area, design, procedure and analysis

The research was based in two peri-urban communities in the eThekwini Municipality, namely Clermont and KwaDabeka. According to the 2011 census (Statistics South Africa 2012), Clermont has a population of 52 075. It covers an area of 6.94 km², with a density of 7500.8 inhabitants/km². KwaDabeka has a population of 52 943. It covers an area of 11.87 km², with a density of 4630.4 inhabitants/km². The population in both communities consists mostly of persons with lower socioeconomic status. The housing varies between informal settlements, government-provided housing, private housing and hostels. The Clermont KwaDabeka area is seen as a relatively mature settlement. Twenty-seven per cent of the population, resident in the area, are under the age of 15. Of the residents, 58% are within the economically active age cohort; however, only 32% are employed. Within the community, 42% use mini-bus or taxis, 42% walk or ride their bicycles. Car ownership is extremely low and only 10% of the population use private vehicles to commute (eThekwini Municipality 2010).

The study followed a convergent mixed methods design (Creswell 2014) with concurrent timing and a qualitative emphasis, with merging of data at the interpretation level. Saturation sampling of CHWs and the ward councillors^4^ in the two communities was employed with a final sample of 53 CHWs and 4 ward councillors. A demographic survey with CHWs, two focus groups with CHWs and semi-structured interviews with four ward councillors were conducted. The following four main areas (with a total of 33 questions) were explored in the survey: (1) biographical information, (2) educational and skills information, (3) job understanding and (4) training.

The focus groups with CHWs included a total of five open exploratory questions (with probes) exploring three main areas: (1) knowledge of their scope and role, (2) perceptions of barriers and enablers to their service delivery and (3) perspectives on their interactions in the community. The interviews with ward councillors comprised seven open-ended questions (with probes) covering three main areas: (1) knowledge of the role CHWs play in the community, (2) perception of possible barriers to and enablers of the service delivery by CHWs and (3) opinion of communities’ perception of the CHW programme.

The focus groups and interviews were digitally recorded with manual recording of fieldwork notes. These were transcribed and data were coded, categorised and organised into themes using NVIVO version 10 via inductive reasoning. With the assistance of a qualified survey developer, surveys were developed online, with information being collated directly into Excel spreadsheets once entered. The data were analysed descriptively and organised into two categories of data, namely nominal (e.g. age and years of experience) and ordinal data (Likert scales regarding their opinion of the CHWs training). Likert scales were analysed to find a central tendency for the opinion of the CHWs about their training and these data were summarised with a median.

### Trustworthiness, reliability and validity

The survey was developed with the assistance of a survey developer, who assisted the researcher in creating the format of the survey in order to capture data as accurately as possible. The digital recordings and field notes were transcribed and audio recordings were validated for accuracy. Other aspects around credibility, transparency and dependability were addressed by the provision of thick descriptions, verbatim quotations, triangulation of method (survey, focus groups and interviews), triangulation of sources (CHWs and ward councillors) and peer debriefing. The first author’s relationship with both groups of CHWs was considered and judgements suspended as part of bracketing by the primary author in an attempt to remain objective. Member checking within the focus groups and interviews was used as a strategy to gain clarity and minimise misinterpretation of the data. Analysis was performed as objectively and accurately as possible by the researcher, and care was taken to ensure the identified bias did not elicit an effect on the study, thereby adding rigor to the study (Yin 2014).

### Ethical considerations

The researcher was granted ethical clearance from a Human and Social Sciences Ethics Committee (HSS/0498/015M) including KZN Provincial Administration Ethics Committee. Permission from the CEO of the two Community Health Centres (CHCs) was also sought. Informed consent was obtained with emphasis on the right to withdraw from the study and the low risk and minimal direct benefit of the study to the CHWs.

## Results

The multiple methods used in this pilot study have assisted in identifying the CHWs and their functioning within the CHW programme in two communities of KZN. Themes are discussed under the following headings: identity and wellness, education and training, knowledge and understanding of roles, and supervision and community education. During the presentation of the results, pseudonyms will be used.

Of the 53 CHWs who participated in the study, all were female, with a mean age of 40. Twenty-two CHWs (40.7%) had a Grade 12 (matriculation) level of education, 26 (48.1%) possessed a Grade 11 and 5 (9.3%) had a Grade 10 level of education. All CHWs expressed that they had been trained as a CHW by completing the National Certificate in home-based care, NQF Level 1. This qualification enables them to work as a health promoter, assistant or health provider, as well as being a health networker within the community development context (South African Qualifications Authority 2012). The mean work experience was 7.3 years (range 1–15 years). Most CHWs volunteered for an average of four years, prior to being officially employed by the DOH as a CHW. This particular group of CHWs had variable work experience. They reported that their experience arose from working in the community and that this was more useful than formal training.

### Identity and wellness

The group of CHWs had different job titles according to different individuals. The ward councillors and CHWs use the title ‘CCG’ (community caregiver), whilst the formal DOH ‘CHW’ (community health worker) has been adopted within policy and legislation. This difference in title could contribute to an apparent lack of clarity regarding job roles and expectations, which will be discussed further in the section ‘knowledge and understanding of roles’. Seventy-eight per cent of the CHWs indicated that they enjoyed their jobs because they helped people. They felt that they were able to improve lives, especially since they were first in line to assist.

‘We are the first person who helps out the community, the nurses are there in the clinic waiting for the person, while the patient is lying on their bed, there is no nurse there.’ (Participant 1, female, CHW)

Other CHWs harboured negative perceptions about their work as a CHW, since they perceived the community to be treating them negatively. As Dudu states:

‘Sometimes because the people are not aware and not knowing what we are doing, they are asking why are we getting money when we are not doing a proper job?’(Participant 2, female, CHW)

The CHWs reported that the community had various expectations of the CHW which constituted duties outside of their prescribed roles. These included expectations of being provided with a meal, requesting for prepaid electricity, being contacted when someone is in labour and expecting massages. As Khanyo states,

I can be angry because it is not my job. (Participant 3, female, CHW)

In addition, some of the CHWs were identified as some other type of healthcare professional, as opposed to a CHW. They were often seen as doctors or social workers.

There was no clear distinction in the programme between ‘employee’ and ‘volunteer’. The CHWs receive a stipend, the amount of which appears to be in direct contravention of the South African labour laws. Full-time employees should receive a monthly salary in accordance with a national standard. At the time of the research, the CHWs were receiving a stipend based on a province-dependent standard. In addition, they were not working under any basic conditions of employment and, therefore, not legally protected. This was also seen nationally in South Africa, within another research report (Lund & Budlender 2009).

Many of the CHWs described symptoms of emotional exhaustion or burnout. These feelings were described as arising from: a lack of supportive supervision, a lack of formal growth pathway within the CHW programme, the receipt of an inadequate stipend, a lack of debriefing following interactions with families experiencing poverty, as well as a dangerous work environment within the communities which they serviced. McIntyre, Mitchell and Ngcwabe (2012) suggest that ‘their stipend might be less worrying to CHWs if there were … definite career paths stemming from CHW and real opportunities to pursue them’ (p. 4).

The CHW to community population ratio at the time of the study was 1:4074, in other words seven times more than the recommended amount of 1:500 (Singh & Sachs 2013). This can be a clear reason for the CHWs being overworked and thus experiencing vocational exhaustion. Burnout has been documented in international literature as affecting healthcare workers in general, and especially community mental health workers (Salyers et al. 2013; Tripathy, Geol & Kumar 2016). They are observed as being more likely to have depression and mental health problems than other members of the healthcare team (Silva & Menezes 2008).

### Education and training

The training for formal qualification as a CHW requires an entrance requirement of matriculation. There is thus a disjuncture between what is possible given the CHWs’ education levels and the feedback received during data collection. Nationally, CHWs have low levels of education (Jinabhai, Marcus & Chapona 2015). However, ward councillor Sandile stated that despite very few of the CHWs having achieved Grade 12, many of them were able to do what people with a Grade 12 education could not do because of their passion and commitment:

‘A CCG member it needs a person who has understanding, who is going to love their job, the person who understands people because you may find that you have a Grade 12 but you won’t go and wash a person who is very sick. Some people don’t have the patience to go and take medication for another person is very sick you understand.’ (Participant 20, male, ward councillor)

Globally, in an examination of selection and training processes in the intervention literature focusing on the role development of CHW, O’Brien et al. (2009) identified ‘a wide variation in the length and content of CHW training … in the reviewed studies’ (p. 262). This is consistent with the CHWs as there is a complexity of training protocols issued through the various DOH centres. Since CHWs are not all employed at the same time, 28% have had significantly more training by a variety of organisations (which include DOH, non-governmental organisations and the like).

Within this study, on average, the CHWs spend 5–10 days in training per month. Of the participants, 7% considered their training as average and that they did not have the necessary skills to perform their jobs. The remaining 93% believed that they did have the necessary skills and that the training was good. Their perception of personal competence is not aligned to the rapid appraisal of CHWs nationally, where CHWs were seen as inadequately prepared and ‘limited by competencies created through legacy vertical programmes’ (Jinabhai et al. 2015:2).

However, when questions were used in the survey to validate the training and knowledge of the CHWs, there appeared to be discrepancies. An example is that 100% of respondents were able to describe what physically happens with the body when someone has a cerebro-vascular accident (CVA)^5^ or stroke; however, only 7% were able to identify what causes a CVA or stroke. Of the group, 94% identified further areas within which they required training. These included wheelchair training (the different types, being able to identify when someone needs a wheelchair and wheelchair transfers), knowledge on paediatric development, basic nursing skills, dementia training, mental health training, disability, health promotion, counselling skills, prescription of exercises, immunisations, prevention of mother to child transmission, tuberculosis, health education, maternal health, diabetes and CVA or stroke (aetiology and interventions).

Currently, there appears to be no formal growth pathway for CHWs. This absence contributes to CHWs desiring to seek alternative means of qualifications, for example in nursing. The CHW programme is an essential contribution to the PHC service and the lack of an upward mobility path, standardised payment method and payment amount, and recognition of experience for the CHWs could be detrimental to the long-term sustainability of the programme. Tripathy et al. (2016) noted that fewer career development opportunities were associated with individual level de-motivators among CHWs in rural health facilities in India. In South Africa, the need was established to, through education, enable CHWs to develop a career path (Jinabhai et al. 2015). All councillors indicated that there should be a career progression for CHWs’ careers and that the CHWs should receive training so they could progress to more formal employment levels.

### Knowledge and understanding of roles

All CHWs in the country should be trained with the PHC training package, which identifies 12 roles that are to be performed by the CHWs working in PHC (DOH 2011a). These include home-based care, counselling, support and stress relief, health promotion and education at a household level, referral to relevant departments, initiative and support home-based projects, liaison between DOH and the community, mobilisation against diseases and poor health through campaigns, word of mouth, etc., directly supervised treatment support (DOTS), screening of health-related clinic cards for compliance or default, assessment of health status for all family members and giving advice, weighing infants and babies and recording in ‘Road to Health’ card and providing prevention of mother to child transmission of HIV/AIDS (DOH 2011a). Reportedly, all of the CHWs were trained according to the same norms and standards. However, of the group of 53 CHWs in this study, 30% of the CHWs identified three roles, 20% identified four roles, 17% identified five roles, 17% identified six roles, 11% identified seven roles, 3% identified nine roles and 2% identified 10 roles with none of the CHWs being able to identify all 12 roles. CHWs mostly identified home-based care as their most frequent task, whilst weighing infants and babies and recording the weight on a ‘Road to Health’ card was the least performed task.

Notwithstanding the naming of the roles, CHWs were asked to identify their job tasks. They noted the following as part of their job descriptions: collecting information from the community, door-to-door visits to assess needs, health education and training, encouraging family planning, encouraging medication compliance, encouraging mothers to immunise their children, establishing community profiles, making the family happy, referral to other DOH departments, calling ambulances for sick people, educating pregnant girls not to smoke ‘Whoonga^6^’ (Grelotti et al. 2014), teaching them to make gardens, teaching them how to cook and teaching the family how to care for their sick relative.

The CHWs iterated that they performed jobs that were outside of their prescribed roles. Seventeen per cent of them however refused to do these. The remaining 93% of respondents, however, described the following additional roles: collection of grants, calling ambulances, assisting in delivery of babies, doing the laundry, taking calls after hours from patients, referrals for food parcels, cleaning of the household, cooking meals, washing or bathing the clients, working in the patient’s garden, taking patients to apply for a grant, working outside of allocated areas and working on the weekend.

### Supervision and community education

Heathe (2011) highlights that systematic supervision is necessary for successful CHW programmes. The CHWs should have clear expectations from their supervisor and be provided with regular support and mentorship. Within the communities studied, there was only one CHW supervisor per CHC site and these CHW supervisors were fulfilling dual roles of supervisor and nurse with the CHC or clinic. Participants felt that minimal supervision and support was being provided, which could create an ineffective system. Also at the time of the study, the NCHWPF did not detail an action plan to establish supportive supervision programmes. Jinabhai et al. (2015) note that there is a national lack of management training, which results in poor CHW supervision and management. The key point strategy for the CHW programme is community education. Swider (2002) identifies that CHW intervention is most effective when implemented within a carefully defined target population and when this population has been educated about the programme to understand the roles and tasks of CHWs. This study revealed that CHWs are not completely aware of their roles in the CHW programme. They thus appear non-adherent to the restrictions in their formal roles and tasks.

Some CHWs had good interactions with the CHCs and resident nurses, with others reporting poor interactions. Poor communication was noted in the referral of clients to the CHC as was required by CHWs, with a report that they would not receive feedback from the nurses regarding these clients that were referred. Anseel and Lievens (2007) highlighted in their study the long-term impact of the feedback environment on job satisfaction. In light of this, the lack of validation derived from a lack of feedback appeared to contribute to poor job satisfaction of the CHWs. Jinabhai et al. (2015) also noted a national lack of back referrals from clinics to the WBPCHOT which include CHWs.

### Issues around stipends and remuneration

One of the most active discussion areas was focused on the stipend received by CHWs. All CHWs reflected negatively on the fact that they earned a meagre stipend whilst they needed to cover their own transport to and from their allocated area of work and that they worked a normal work day of 8 h duration. Some CHWs did not access formal modes of transport and instead walked to and from their allocated area of work. A lack of resources, in particular not having equipment such as masks or gloves, was also raised:

‘But the job is a lot of work but no money, how can you live?’ (Participant 4, female, CHW)

‘It’s hard because some of us are using the small money they give us to take transport to the area we are working. That makes it very hard because there is little money left sometimes.’ (Participant 5, female, CHW)

In addition, the CHWs reported that the community questioned their stipend. All ward councillors agreed that the stipend was an issue of concern for the CHWs. Ward councillor Sbusiso stated that if the issue surrounding the stipend was resolved, it would help to reduce the ‘excuses the CHWs were providing so as to not work’. Councillor Sandile also echoed this sentiment and indicated that if the CHWs received more money, then they would be able to do more. Councillor Bonga identified that the challenge lay in identifying people who were passionate about being a CHW and those that are not just in it for the money.

### Ward councillors’ opinions of the community health worker programme

All ward councillors agreed that in theory the CHW programme was highly necessary, but that the programme needed to be coordinated better. The councillors stated that it was also important to have awareness campaigns so that the community learned about the CHWs’ roles and that there needed to be more education about the programme in the community. Ward councillors Sandile and Bonga reported positive experiences with the CHWs in their wards. They were of the opinion that CHWs appeared to enjoy their jobs, loved their communities and felt that they were making an impact because of their compassion for the communities they serve. Both of these ward councillors reported a mutual relationship between CHWs and themselves in order to ensure service delivery.

‘As government officials we can’t be on the ground we have to make things happen we have to come with solutions but we can’t come with solutions if there are no field workers people who go out in the community find out exactly what is happening and then come back to report.’ (Bonga, Participant 21, male, ward councillor)

Ward councillor Sbusiso stated that his experiences with the CHWs had been difficult and disturbing. He described the CHWs as

‘ineffective, lazy, not wanting to do the best they could, not wanting to work, dragging their feet, working only for money and not for the passion of the job and thus having lost their commitment to the job.’ (Participant 22, male, ward councillor)

He summarises it as follows:

‘So, in a nutshell it is difficult to work with them they have lost the commitment that is expected of them right now. I don’t even know where they are, maybe they are sleeping at home, and maybe some of them are in the field.’ (Participant 22, male, ward councillor)

The councillor went on to say that he questioned what CHWs actually did in the field as there was a disparity between what was reported to him and what he observed. The CHWs were said to rarely report to the councillor, when requested, and did not hand in the relevant documents when required.

These findings show the profile of the CHWs studied as well as provide deeper insight into their knowledge of job roles, expectations from the community, training and supervision and feelings of working as CHWs. It is clear that some CHWs have positive working experiences, whilst others have negative working experiences.

## Conclusion

This article has provided an overview of the CHWs within two peri-urban communities of KZN. The study has identified implications for the human resources for health in South Africa. It highlights violations of a quality and equitable health service, thus posing serious risks to the implementation of the PHC re-engineering to provide Universal Health Coverage.

This study has numerous implications for policy and practice of the essential PHC service. The pillars on which the National Health Insurance is being built require an efficient CHW team, to enable home-based care and early identification of illness and disease, thus limiting future disability. In addition, the service should strive for health promotion and disease prevention, which will create greater thriving and healthy communities, who are living towards their full potential.

### Recommendations

Kautzky and Tollman (2008) call for a ‘careful consideration of the skills and competencies needed in the PHC system’ (p. 27) as critical to its success. Although this study focused only on the CHWs in two communities, these recommendations may be pertinent within other communities as well as in the system as a whole.

#### Evaluation and monitoring

- Systems should include feedback and discussion forums, where challenges experienced by both the CHW and the community are addressed. This should include ward councillors and CHW supervisors aiming towards a climate of communication via the War Rooms^7^ where the communities are also involved.
- Each CHW programme requires review according to the provincial and national strategies and standards, to assess their efficacy.
- There should be feedback provided to the CHWs when they refer clients to the CHCs. This will improve the case management of the clients as well as improve job satisfaction since the CHWs will have an improved understanding of the ongoing health service provided to their clients.

#### Supervision

- As discussed, this study had only one supervisor per site responsible for the CHWs from each CHC. Because of the high work load and support required, it is unsustainable and thus at least two supervisors should be appointed.
- CHW supervisors should not be required to hold dual responsibilities of both nursing and supervision. Their role needs to be dedicated to the training, supervision and evaluation of the CHW programme.
- Supportive supervision should be thoroughly outlined and include elements of record reviews, observations, performance monitoring, constructive feedback, provider participation, problem solving and focused education (Bosch-Capblanch & Garner 2008).
- Supervision needs to be supportive and address issues of burnout and compassion fatigue that may be experienced by the CHW. There should be a protocol where once this has been identified, a clear pathway of intervention is outlined. This needs to be implemented and followed up.
- There should be clear distinction between CHWs experiencing symptoms of burnout and exhaustion and those that have a poor attitude towards service delivery. An appropriate intervention strategy is required for those with a poor attitude and poor service implementation.

#### Community awareness

- The community War Rooms should be used as avenues to discuss the title, role and scope of the CHWs. The ward councillors, however, should be at the helm of ensuring that carry-through occurs.
- The DOH should ensure that the number of CHWs is in alignment with the ratio of CHW to population 1:500 (Singh & Sachs 2013). When the apparent work load of the CHWs is addressed in this way, it may have a positive effect on the quality of service provision to the community.

#### Policy development and evaluation

- The training of the CHWs should be incorporated into the EPWP training strategy, which will enable CHWs to obtain a formal qualification which is aligned to a national standard. This will also ensure that each CHW receives the full training required, as opposed to ad-hoc sessions based upon time of acceptance into the CHW programme.
- There should be a growth pathway for CHWs, to ensure that persons with experience can achieve higher levels of employment and thus also mentor the newer applicants in the programme.
- The status of the CHWs needs to be aligned to the South African Labour Laws regarding the basic conditions in the employment act. A clear decision must be taken on the status of employment, namely volunteer or full-time employee. There should be an investigation into the remuneration package received by the CHW and its lack of alignment to the South African Labour Laws.
- Further studies should include community perspectives and perspectives of the CHW supervisors to obtain holistic information regarding the functioning of the programme.

## Footnotes

1 This refers to individuals who are unable to afford private healthcare insurance.

2 These are generalist services that are taken out to the community. They are an add-on service to PHC clinics. Locations are allocated according to which wards the clinic providing the outreach service is responsible for delivering services to.

3 This is a formal system used to describe qualifications.

4 These are members of the community, elected as members of the Ward Committee to represent the community at council meetings.

5 The sudden death of some brain cells due to lack of oxygen when the blood flow to the brain is impaired by blockage or rupture of an artery to the brain. A CVA is also referred to as a stroke.

6 Whoonga is a South African drug cocktail that contains illicit drugs and HIV antiretroviral (ARV) medication.

7 War Rooms are a government initiative indicating its desire to work with non-governmental organisations (NGOs), communities and other sectors to ensure quality service delivery. This promise of action should improve integration and coordination, reduce duplication and inefficiencies, clearly define roles and utilise existing resources optimally to service people closer to where they live (Mebalo 2014).
